# Complete chloroplast genome of *Tricyrtis xianjuensis* Li, Chen & Ma 2014 (Liliaceae): a species endemic to Zhejiang province, China

**DOI:** 10.1080/23802359.2023.2301021

**Published:** 2024-01-08

**Authors:** Leqin Huang, Zhenyu Lu, Junfeng Wang, Honghua Bao, Huijuan Zhang, Ming Jiang

**Affiliations:** a College of Life Sciences, Taizhou University, Taizhou, China; b Scientific Research Management Center, East China Medicinal Botanical Garden, Lishui, China; c Taizhou Municipal Ecology and Environment Bureau, Taizhou, China

**Keywords:** *Tricyrtis xianjuensis*, rare species, chloroplast genome, phylogenetic analysis

## Abstract

*Tricyrtis xianjuensis* Li, Chen & Ma 2014 is a rare and endangered species endemic to Zhejiang province, with fewer than 200 individuals in the wild. In our present study, the complete chloroplast genome of *T. xianjuensis* was assembled by using high-throughput sequencing data, and its genomic features were described and comparative genomic analyses within Liliaceae family were performed. The complete chloroplast genome of *T. xianjuensis* was 155,748 bp in length, exhibiting a GC content of 37.3%. This genome structure contained two inverted repeats (IRs), as well as a small single-copy (SSC) and a large single-copy (LSC) region. The IR region measured 26,371 bp, while the SSC and LSC regions were 17,729 bp and 85,277 bp in length, respectively. A total of 137 genes were identified, including 85 protein-coding genes, 38 tRNA genes, eight rRNA genes, and six pseudogenes. Phylogenic analysis revealed *T. xianjuensis* shared a clade with *T. formosana* Baker 1879 and *T. macropoda* Miq. 1867, with a support rate of 100%. The assembly and analysis of *T. xianjuensis* chloroplast genome provided an insight into further studies on the conservation genetics of this endangered species.

## Introduction

Chloroplasts are organelles that perform diverse functions, including photosynthesis, as well as biosynthesis of phytohormones, starch, lipids, and vitamins (Chen et al. 2018). The chloroplast genome contains genes necessary for synthesis of proteins involved in photosynthesis and metabolic processes, and they are widely used in plant diversity, evolution, and species identification (Daniell et al. 2016). The genus *Tricyrtis*, classified within family Liliaceae, is a small-sized group comprising approximately 18 species, and they are predominantly distributed from Himalayas to East Asia (Wu et al. 2000; Sophia and Jury 2012). There are about 10 *Tricyrtis* species distributed in China, including *T. macropoda* Miq 1867, *T. pilosa* Wall. 1826, and *T. xianjuensis* Li, Chen & Ma 2014. *T. macropoda* and *T. pilosa* are widely distributed across many provinces in China (Wu et al. 2000), while *T. xianjuensis* has a limited distribution range and is only found in Shenxianju Mountain, Xianju County, Zhejiang Province (2014). By searching NCBI database, we found two species, *T. macropoda* and *T. formosana* Baker 1879, for which the chloroplast genomes have been assembled. Currently, there is only one published report available regarding the research on *T. xianjuensis* (Ma et al. 2014). To clarify the sequence characteristics, gene composition, and taxonomic relationship, the chloroplast genome of *T. xianjuensis* was assembled, and a phylogenetic tree was constructed, which could serve as a foundation for further investigation on conservation genetics.

## Materials and methods

### Plant sampling

**Figure 1.**
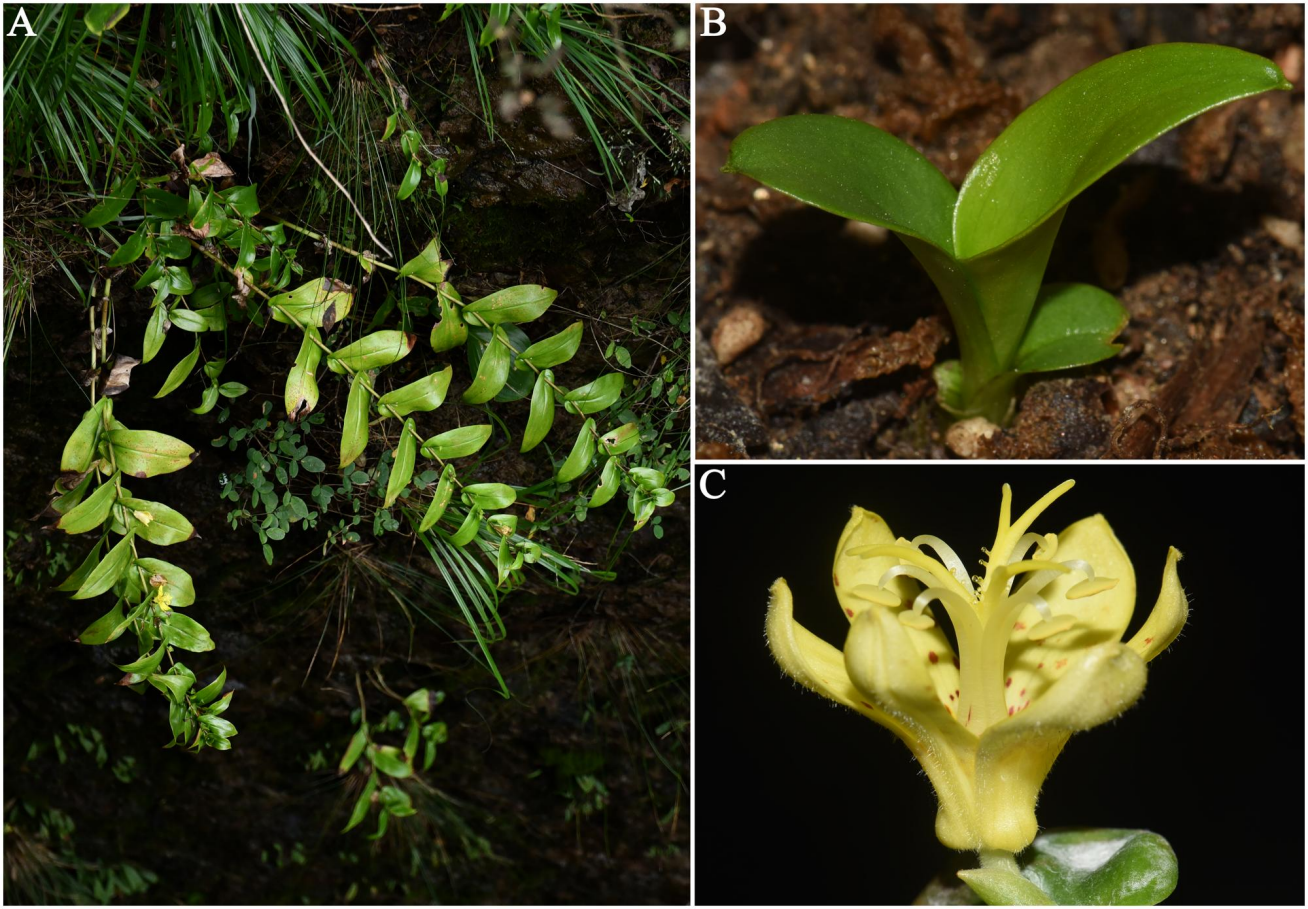
*Tricyrtis xianjuensis* Li, Chen & Ma 2014. (A) Habitat; (B) seedling; (C) flower. All the photos were taken by Ming Jiang. *Tricyrtis xianjuensis* is a perennial herb up to 70 cm, with short rhizomes, ascending stems, alternate leaves, and yellow flowers. Flowering period of this species occurs between September and early October, while fruiting in October.

Leaves were collected from Shenxianju Mountain (28°41′19″, 120°37′8″, 865 m), Xianju County, Zhejiang, China (Figure 1). Leaves were kept in a foam box filled with ice packs and taken to our laboratory. A voucher specimen CHS20210038 was deposited in Molecular Biology Innovation Laboratory at Taizhou University (Ming Jiang, jiangming1973@139.com).

### DNA isolation, sequencing, assembling, and annotation

To isolate genomic DNA, we utilized the CTAB method (Doyle and Doyle 1987). We constructed a genomic DNA library and performed sequencing by the Illumina Hiseq X Ten System. Low-quality sequences were filtered using NGSQCToolkit v2.3.3 (Patel and Jain 2012). The chloroplast genome was assembled by NOVOPlasty with default parameters (Dierckxsens et al. 2017), and *matK* of *T. macropoda* (KU303884) was utilized as a seed. A map of *T. xianjuensis* chloroplast genome was generated using CPGview (http://www.1kmpg.cn/cpgview/) (Liu et al. 2023). The simple sequence repeats (SSRs) were identified using MISA software (Allen et al. 2011).

### Phylogenetic analysis

A total of 25 chloroplast genomes from species within Liliaceae family were retrieved from NCBI, and the sequences of *Croomia japonica* Miq. 1865 and *C. pauciflora* (Nutt.) Torr. 1840 were also downloaded as the outgroup. The genomes were aligned using MAFFT v7.450 (Katoh and Standley 2013), and a phylogenetic tree was constructed using the maximum-likelihood method by PhyML 3.1 (Guindon et al. 2010), with the best-fit substitution model of GTR + R.

## Results

The high-throughput sequencing resulted in 3.79 G raw data, consisting of 12,618,427 reads, and the average sequencing depth was 853.83 (Figure S1). After removing low-quality reads, the clean data accounted for 3.75 G, comprising 12,484,278 high-quality reads. The complete chloroplast genome of *T. xianjuensis* was 155,748 bp in length, with a GC content of 37.3%. The genome structure included two inverted repeats (IRs), a small single-copy (SSC) region, and a large single-copy (LSC) region. The IR was 26,371 bp in length, while the SSC and LSC were 17,729 bp and 85,277 bp, respectively. The sequence of *T. xianjuensis* has been submitted to NCBI with the accession number of OR526424.

**Figure 2.**
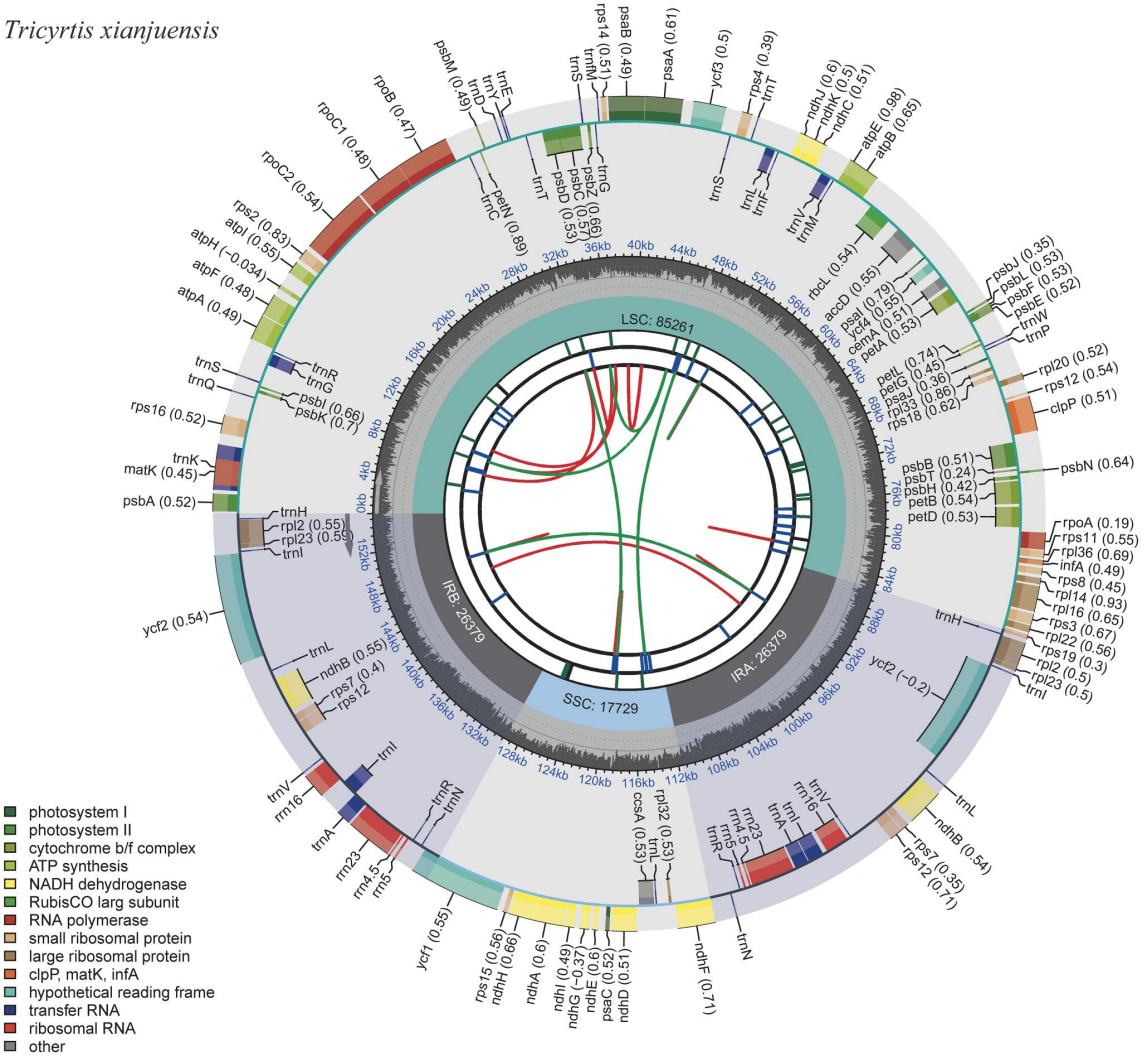
The chloroplast genome map of *Tricyrtis xianjuensis*. The map encompasses six tracks that depict various features of the genome. Starting from the center, the first track displays dispersed repeats, consisting of both direct and palindromic repeats, which are delineated by red and green arcs. The second track highlights long tandem repeats represented by short blue bars. In the third track, short tandem repeats or microsatellite sequences are illustrated as colored bars. Each color corresponds to a specific type of repeat, and accompanying descriptions provide valuable information about the characteristics of each repeat type. The colors and their respective repeat types are as follows: black: c (complex repeat); green: p1 (repeat unit size = 1); yellow: p2 (repeat unit size = 2); purple: p3 (repeat unit size = 3); blue: p4 (repeat unit size = 4); orange: p5 (repeat unit size = 5); red: p6 (repeat unit size = 6). The chloroplast genome contains an LSC region, an SSC region, and two IR regions, and they are shown on the fourth track. The GC content along the genome is shown on the fifth track. Genes within the genome visualization are meticulously color-coded based on their functional classification. The transcription directions of the inner genes are represented in a clockwise manner, while the outer genes are shown in an anticlockwise orientation. To assist with interpretation, the key for gene functional classification is provided in the bottom left corner of the visualization.

Gene annotation revealed a total of 137 genes on *T. xianjuensis* chloroplast genome, which consisted of 85 protein-coding genes, 38 tRNAs, eight rRNAs, and six pseudogenes (Figure 2). A small proportion of genes exhibited two copies, with 11 protein-coding genes (*ndhB*, *rpl2*, *rpl23*, *rpoC1*, *rps7*, *rps12*, *rps19*, *ycf1*, *ycf2*, *ycf15*, and *ycf68*) and eight tRNAs (*trnA-UGC*, *trnH-GUG*, *trnI-CAU*, *trnI-GAU*, *trnL-CAA*, *trnN-GUU*, *trnR-ACG*, and *trnV-GAC*). Thirteen genes possessed one or two introns. Among them, *rps16*, *atpF*, *rpoC1*, *petB*, *petD*, *rpl16*, *rpl2*, *ndhB* (two copies), and *rpl2* (two copies) contained one intron each, while *ycf3* and *clpP* held two introns each (Figure S2). In addition to the cis-splicing genes mentioned above, there was also one trans-splicing gene, *rps12*, present in the genome (Figure S3). Moreover, a total of six pseudogenes were identified, including ψ*ycf15* (two copies), ψ*ycf68* (two copies), ψ*ycf1*, and ψ*rps19*. A total of 33 SSRs were predicted, and all were found to be mononucleotides.

**Figure 3.**
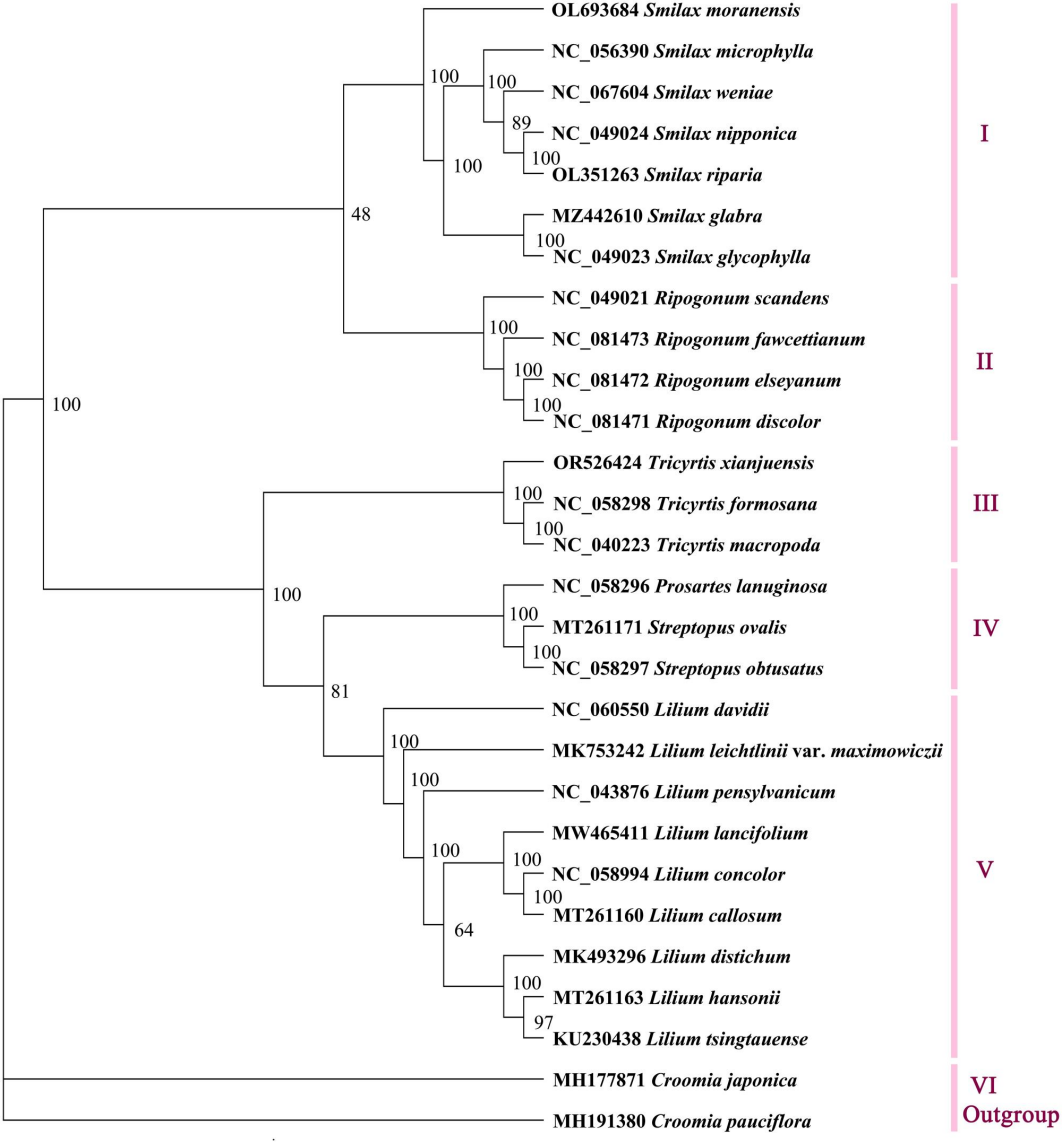
The maximum-likelihood tree based on complete chloroplast genome sequences of *Tricyrtis xianjuensis* and 25 species of family Liliaceae, with *Croomia japonica* and *C. pauciflora* as the outgroup species. The numbers next to the nodes are bootstrap support values. NCBI accession numbers of each genome are shown in the figure. The following sequences were used: *Croomia pauciflora* MH191380 (Lu et al. 2018), *C. japonica* MH177871 (Lu et al. 2018), *Smilax moranensis* OL693684 (Ji et al. 2022), *S. microphylla* NC_056390 (Wu et al. 2021), *S. weniae* NC_067604 (Feng et al. 2022), *S. nipponica* NC_049024, *S. riparia* OL351263, *S. glabra* MZ442610, *S. glycophylla* NC_049023 (Do et al. 2020), *Ripogonum scandens* NC_049021 (Do et al. 2020), *R. fawcettianum* NC_081473, *R. elseyanum* NC_081472, *R. discolor* NC_081471, *Tricyrtis formosana* NC_058298, *T. macropoda* NC_040223 (Wang et al. 2018), *Prosartes lanuginose* NC_058296, *Streptopus ovalis* MT261171 (Do et al. 2020), *S. obtusatus* NC_058297, *Lilium davidii* NC_060550 (Li et al. 2021), *L. leichtlinii* var. *maximowiczii* MK753242, *L. pensylvanicum* NC_043876 (Ramekar et al. 2019), *L. lancifolium* MW465411, *L. concolor* NC_058994, *L. callosum* MT261160 (Do et al. 2020), *L. distichum* MK493296, *L. hansonii* MT261163 (Do et al. 2020), and *L. tsingtauense* KU230438 (Song et al. 2016).

A phylogenetic tree was constructed using full length chloroplast genome sequences. The results showed that the 29 sequences could be divided into six major groups, namely I, II, III, IV, V, and VI. The phylogenetic analysis indicated that species from the same genus clustered together, such as *Smilax* (I), *Ripogonum* (II), *Tricyrtis* (III), and *Lilium* (V). However, there existed an exception where *Prosartes lanuginosa* (Michx.) D.Don 1839 formed a group (IV) with *Streptopus ovalis* (Ohwi) F.T.Wang & Y.C.Tang 1978 and *S. obtusatus* Fassett 1935 (Figure 3). The results revealed a close relationship between the three *Tricyrtis* species; *T. macropoda* and *T. formosana* first clustered together, then formed a group with *T. xianjuensis*, supported by a 100% confidence rate.

## Discussion and conclusions

Chloroplast genomes are highly conserved in both structure and gene content, and they are regarded as useful molecular tools for genetic diversity and evolution studies (Perumal et al. 2021). The chloroplast genome of *T. xianjuensis* was not assembled and analyzed, which hinders further investigation into its conservation genetics. It was observed that the length of the chloroplast genomes in *Tricyrtis* is highly conserved. The full length of *T. formosana* (NC_058298.1) is 156,018 bp, only 270 bp longer than that of *T. xianjuensis*. Similarly, the complete length of *T. macropoda* (NC_040223.1) (Wang et al. 2018) is 155,778 bp, merely 22 bp longer than that of *T. xianjuensis*. However, compared to *matK* gene and *rps16* intron, which were previously used for phylogenetic and evolutionary analyses of *Tricyrtis* species, the chloroplast genome offers a greater number of variable sites (Sophia and Jury 2012).

*T. xianjuensis* demonstrated a duplication event involving the *rps19* gene, with one copy being a pseudogene. The pseudogene was characterized by a 3′ end deletion, triggered by the IRb/LSC boundary located within this copy. Pseudogenization of *rps19* caused by the IRb/LSC boundary has been documented in multiple plant species, including *Dianthus superbus* var. *longicalyncinus* (Maxim.) Will. 1899, *Decaisnea insignis* (Griff.) Hook. fil. & Thomson 1854, and *Cerasus humilis* (Bunge) S. Ya. Sokolov 1954 (Raman and Park 2015; Li et al. 2017; Mu et al. 2018). The *ycf68* gene was present in two copies in *T. xianjuensis*. However, both copies were pseudogenes with several internal stop codons within their sequences. Similar occurrences have been observed in other plants such as *Angiopteris evecta* (G.Forst.) Hoffm. 1793, several *Mammillaria* species, and *Zephyranthes phycelloides* (Herb.) Nic. García 2019 (Roper et al. 2007; Solórzano et al. 2019; Contreras-Díaz et al. 2022).

In conclusion, the complete chloroplast genome of *T. xianjuensis* was 155,748 bp with a total of 137 genes, and phylogenetic analysis revealed *T. xianjuensis* shared a clade with *T. formosana* and *T. macropoda*. Assembly and sequence analysis of the complete chloroplast genome sequence of *T. xianjuensis* provided insights into population genetics and biodiversity studies for this rare species in the future.

## Supplementary Material

Supplemental Material

## Data Availability

The data that support the findings of this study are openly available in GenBank of NCBI at https://www.ncbi.nlm.nih.gov/nuccore/ OR526424. The associated BioProject, SRA, and Bio-Sample numbers are PRJNA1025334, SRR26334644, and SAMN37721183, respectively.
