# Supplementary material for: Complete chloroplast genome of Tricyrtis xianjuensis Li, Chen & Ma 2014 (Liliaceae): a species endemic to Zhejiang province, China
Source: Mitochondrial DNA B Resour. 2024 Jan 8;9(1):60–5. doi: 10.1080/23802359.2023.2301021 (PMC10776049; doi:10.1080/23802359.2023.2301021)
Supplement: Supplemental Material [file TMDN_A_2301021_SM2743.docx]

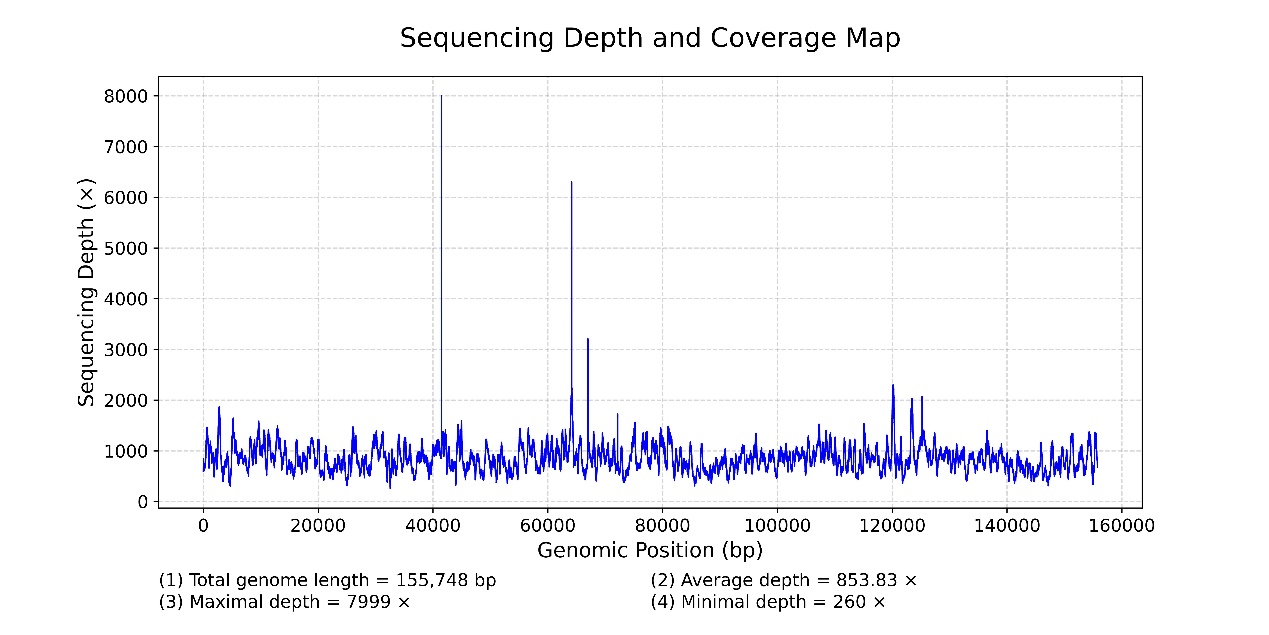


**Figure S1** Coverage depth figure of the *Tricyrtis xianjuensis* chloroplast genome. The horizontal coordinate is the position of the chloroplast genome, and the vertical coordinate is the sequencing depth.





**Figure S2** Schematic map of the cis-splicing genes in the *Tricyrtis xianjuensis* chloroplast genome.





**Figure S3** Schematic map of the trans-splicing gene *rps12* in *Tricyrtis xianjuensis* chloroplast genome.
